# Identification of a plasma metabolomic signature of thrombotic myocardial infarction that is distinct from non-thrombotic myocardial infarction and stable coronary artery disease

**DOI:** 10.1371/journal.pone.0175591

**Published:** 2017-04-17

**Authors:** Andrew P. DeFilippis, Patrick J. Trainor, Bradford G. Hill, Alok R. Amraotkar, Shesh N. Rai, Glenn A. Hirsch, Eric C. Rouchka, Aruni Bhatnagar

**Affiliations:** 1 Department of Medicine, Division of Cardiovascular Medicine, Diabetes and Obesity Center, University of Louisville, Louisville, Kentucky, United States of America; 2 Johns Hopkins University, Baltimore, Maryland, United States of America; 3 Department of Medicine, Division of Bioinformatics, University of Louisville, Louisville, Kentucky, United States of America; 4 Department of Bioinformatics and Biostatistics, University of Louisville, Louisville, Kentucky, United States of America; 5 Department of Computer Engineering and Computer Science, University of Louisville, Louisville, Kentucky, United States of America; Emory University, UNITED STATES

## Abstract

**Aims:**

Current non-invasive diagnostics for acute myocardial infarction (MI) identify myocardial necrosis rather than the primary cause and therapeutic target—plaque disruption and resultant thrombosis. The aim of this study was to identify changes specific to plaque disruption and pathological thrombosis that are distinct from acute myocardial necrosis.

**Methods and results:**

We quantified 1,032 plasma metabolites by mass spectrometry in 11 thrombotic MI, 12 non-thrombotic MI, and 15 stable coronary artery disease (CAD) subjects at two acute phase (time of catheterization [T0], six hours [T6]) and one quiescent (>3 months follow-up) time points. A statistical classifier was constructed utilizing baseline (T0) abundances of a parsimonious set of 17 qualifying metabolites. Qualifying metabolites were those that demonstrated a significant change between the quiescent phase and the acute phase and that were distinct from any change seen in non-thrombotic MI or stable CAD subjects. Classifier performance as estimated by 10-fold cross-validation was suggestive of high sensitivity and specificity for differentiating thrombotic from non-thrombotic MI and stable CAD subjects at presentation.

Nineteen metabolites demonstrated an intra-subject change from time of acute thrombotic MI presentation to the quiescent state that was distinct from any change measured in both the non-thrombotic MI and stable CAD subjects undergoing cardiac catheterization over the same time course (false discovery rate <5%).

**Conclusions:**

We have identified a candidate metabolic signature that differentiates acute thrombotic MI from quiescent state after MI, from acute non-thrombotic MI, and from stable CAD. Further validation of these metabolites is warranted given their potential as diagnostic biomarkers and novel therapeutic targets for the prevention or treatment of acute MI.

## Introduction

Acute myocardial infarction (MI) remains a leading cause of death worldwide. Although plaque rupture and the ensuing coronary thrombosis are hallmarks of myocardial infarction (MI), many non-thrombotic causes of myocardial infarction / necrosis are known (i.e., demand ischemia, direct myocardial toxins, etc).[1] Although troponin levels are sensitive and specific for myocardial necrosis, they do not provide any information on the cause of myocardial infarction (necrosis). While international guidelines recognize different types of MI, i.e., thrombotic (type 1) and non-thrombotic (type 2) MI, no diagnostic criteria exist for differentiating between these types of MI, which require different treatment strategies.[2] One recent study of all troponin tests ordered by treating physicians in a hospital system found 42% of troponin tests to be positive; 29% were secondary to non-thrombotic (type 2) etiologies compared with 13% from an acute thrombotic MI diagnosis.[3] Mortality was 59% at 3.2 years in the patients with non-thrombotic (type 2) troponin elevations.[3] Furthermore, while circulating levels of troponin are specific indicators of myocardial necrosis, the levels of these proteins often do not increase for several hours after the start of an acute MI.[4] Therefore, current diagnostic criteria for acute MI are unable to delineate the cause of acute MI and often fail to confirm the diagnosis before the induction of irreversible myocardial necrosis, even with modern “super sensitive” cardiac troponin assays.[5]

The limitations to existing diagnostic strategies are highlighted by the fact that 70% of the ~8 million US patients presenting to the hospital with chest pain are diagnosed with benign chest pain, at an annual cost of $10 billion; however, 2–5% of these discharges are subsequently found to have an acute MI with a worse prognosis than those correctly diagnosed on the initial encounter.[6–8] Furthermore, although patients with thrombotic MI derive substantial benefit from current anticoagulant and antiplatelet therapies, many patients with non-thrombotic MI (e.g., type 2 or demand ischemia) are exposed to the bleeding risk of these therapies without potential clinical benefit.

Due to its unpredictable nature, complex etiology, and inability to produce or detect spontaneous coronary thrombosis in an animal model, the pathophysiology of thrombotic MI has not been studied directly. The mechanisms leading to plaque disruption and those that determine the nature and the extent of the ensuing thrombotic responses remain unclear. Nearly 80% of plaque ruptures are “healed” via a controlled hemostatic response; these do not result in occlusive coronary thrombosis.[9] Conversely, 27–35% of cases of acute thrombotic STEMI or cardiac death are triggered by minimal changes in plaque morphology (e.g., erosion of the endothelial layer), without the exposure of the necrotic core of the plaque to luminal blood.[10–13] The determinants of a “pathological” versus “healing” or “homeostatic” thrombotic response to plaque disruption remain unknown. However, via novel study designs and analytical approaches, it may be possible to identify specific changes accompanying plaque disruption, thrombus formation, and acute MI. Metabolomics, the study of small molecule intermediates and products of metabolism, could provide a comprehensive assessment of the state of a biological system at the time of sampling, and therefore has the ability to capture dynamic changes such as plaque disruption and resultant thrombosis.[14]

In patients presenting with symptoms concerning for acute MI, the ability to identify a thrombotic MI prior to substantial myocardial necrosis (troponin release) and / or to quickly distinguish thrombotic from non-thrombotic MI and stable coronary artery disease (CAD) would improve triaging of patients for expedited administration of appropriate therapies resulting in improved outcomes at lower cost. Such a distinction would allow for more judicious use of anticoagulant therapy, thereby improving patient safety without sacrificing efficacy. Furthermore, the identification of metabolites that change at the time of and are specific to acute thrombotic MI could lead to a new understanding of the mechanisms underlying plaque disruption that lead to coronary thrombosis and ischemia rather than a “homeostatic” response without immediate clinical consequence. Such understanding could lead to the development of novel therapies for one of the world’s most common cause of death—acute MI. Therefore, in this study we tested the hypothesis that changes in circulating metabolites at the time of acute thrombotic MI are distinct from changes at the time of acute non-thrombotic MI and diagnosis of stable CAD.

## Methods

### Study approval and enrollment population

Following institutional review board approval (University of Lousiville), subjects with suspected acute MI and stable CAD, scheduled for cardiac catheterization, were recruited from two hospitals in Louisville, Kentucky between March 2012 and August 2013 and followed prospectively. All subjects provided written informed consent. Additional details of the enrollment criterion are noted in the supplement (S1 File).

### Study cohort

Given the lack of accepted guidelines for differentiating thrombotic and non-thrombotic MI, we developed novel and stringent criteria (Table 1) to eliminate borderline cases from our analysis. This was done to limit confounding factors due to misclassification. These criteria expand upon those previously proposed by our group.[15, 16] Additional details on study criteria for classification as thrombotic MI, non-thrombotic, and stable CAD are provided in the supplement (S1 File).

**Table 1:** Study phenotype criteria.

Study Phenotype	Criteria			
	Troponin (ng/ml)	Histology	Presentation	Blinded Angiographic Assessment
**Thrombotic** **MI (n = 11)**	**Ortho Vitros 5600 Assay:** “Peak” Troponin Level **>0.12** **Beckman Access:** “Peak” Troponin Level **>0.5-and-** >30% increase in troponin from T0 to T6	Histologically confirmed coronary thrombus 0–4 days old by blinded pathological assessment.	Clinical presentation consistent with WHF/ECC/ACC/AHA Universal definition of AMI	Stenosis of 50–100% in the vessel where thrombus was recovered and absence of dissection.
**Non-Thrombotic** **MI (n = 12)**	**Ortho Vitros 5600 Assay:** “Peak” Troponin Level **>0.12** **Beckman Access:** “Peak” Troponin Level **>0.5** **-and-** >30% increase in troponin from T0 to T6	No histologically confirmed thrombus recovered.	Clinical presentation consistent with WHF/ECC/ACC/AHA Universal definition of AMI	Satisfies ALL 5 criteria below, in all vessels: 1. ≤50% stenosis 2. No filling defect 3. Simple Ambrose lesion morphology 4. TIMI flow = 3 5. TIMI MPG = 3
**Stable CAD (n = 15)**	**Ortho Vitros 5600 Assay:** “Peak” Troponin Level **<0.035** **Beckman Access:** “Peak” Troponin Level **<0.04**		Elective coronary angiogram History of CVD: CABG, PCI, CVA / TIA, CEA, PAD or AAA procedure–or angiographic criteria	Satisfies ALL criteria below: 1. > 50% stenosis in one or greater epicardial vessel (only required if no history of CVD) 2. TIMI flow = 3 (all vessels) 3. TIMI MPG = 3 (all vessels)

CAD = coronary artery disease, MI = myocardial infarction, TIMI = thrombolysis in myocardial infarction, MPG = myocardial perfusion grade, CABG = coronary artery bypass grafting, PCI = percutaneous coronary intervention, CVA = cerebral vascular accident, TIA = transient ischemic attack, CEA = carotid endarterectomy, PAD = peripheral artery disease, AAA = abdominal aortic aneurysm

### Metabolomics

We performed unbiased discovery metabolomics on plasma samples from each subject at three different time points: 1) time of enrollment (T0) was at the time of cardiac catheterization but prior to any diagnostic (angiogram) or therapeutic intervention; 2) six hours after enrollment (T6), which was also after percutaneous coronary intervention (PCI) for all subjects that had a procedure; and 3) quiescent phase (>3 month follow-up from time of enrollment) in which subjects were in a stable state with no active acute illness. Samples were analyzed using ultra-performance liquid chromatography tandem mass spectrometry (UPLC-MS/MS) in multiple ion modes and gas chromatography mass spectrometry (GC-MS). Reproducibility of measurements has been demonstrated by this laboratory.[17] Instrument variability was determined by calculating the median relative standard deviation for internal standards added to each sample prior to injection into the mass spectrometers. Overall process variability was determined by calculating the median relative standard deviation for all endogenous metabolites (i.e., non-instrument standards) present in a standardized plasma matrix sample. Additional details of metabolomic measurements are provided in the supplement (S1 File).

### Statistical analysis

**Fig 1:**
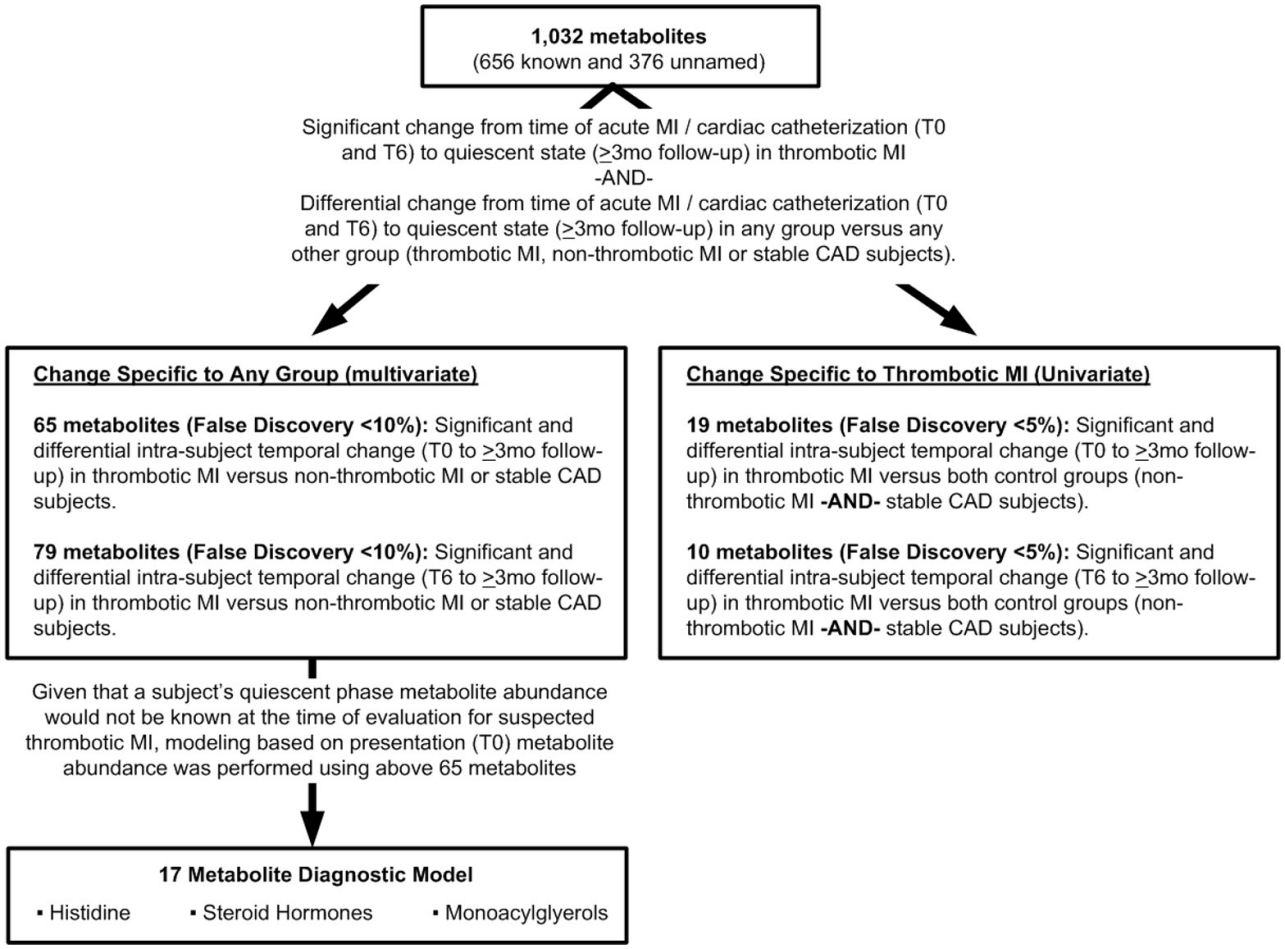
Analytical process.

Frequencies, percentages, and Fisher’s exact test p-values for comparing distributions across groups are reported for categorical characteristics; mean, standard deviation, and appropriate statistical test p-value (one-way ANOVA F-test, Welch’s ANOVA, or Kruskal-Wallis H-test) are reported for comparing the distributions of continuous variables across groups. As metabolites that are specific to thrombotic MI should exhibit temporal change between a quiescent state and the acute thrombotic MI state, and this change should differ from that observed in non-thrombotic MI and stable CAD, a linear model framework was used for testing such hypotheses. The framework consisted of regressing Log_2_-transformed intra-subject temporal fold change on indicators of study group. This allowed for the determination of metabolites with: (1) significant mean intra-subject fold change from acute states (T0 or T6) to the quiescent state (>3 months follow-up) in thrombotic MI, and (2) metabolites with significantly different intra-subject fold change in one versus any of the other groups (acute thrombotic MI, non-thrombotic MI subjects, stable CAD) (Fig 1). Confounding / effect modifications of multiple factors (age, gender, diabetes status, etc.) were controlled for by using subjects as their own control. Mean fold change was defined as the average of intra-subject relative changes in a metabolite within subjects in a group (thrombotic MI, non-thrombotic MI, stable CAD) between two time points (T0 or T6 and quiescent)–therefore each participant served as their own control. Additionally, metabolite changes in thrombotic MI were compared to two control groups (non-thrombotic MI and stable CAD) undergoing cardiac catheterization over the same time course to control for myocardial ischemia / necrosis, underlying disease (atherosclerosis–as opposed to risk factors for atherosclerosis alone), similar procedure / therapeutics, and non-specific acute illness. Thrombotic MI and non-thrombotic MI controls have the same acute disease consequence (myocardial infarction) and have undergone the same procedure (cardiac catheterization), and stable CAD controls have the same underlying disease (atherosclerosis) and also undergo the same procedure as opposed to a naïve “matching” on risk factors for a disease state.

Given the large number of hypotheses tested (1,032 metabolites), q-values for preserving the False Discovery Rate (FDR) at or below a given threshold were reported[18] for determining significance rather than classical p-values. A q-value of 0.05 corresponds to 95 out of the 100 metabolites deemed significant being true positives on average. In this study, two separate significance levels were utilized for assessing statistical significance. A stringent FDR cutoff of 5% was utilized to identify individual metabolites that demonstrated a significant change from acute to quiescent state in thrombotic MI subjects that was distinct from both non-thrombotic MI and stable CAD. To limit the number of metabolites to be evaluated in multivariate analyses, while preserving the ability to capture the effect of metabolites that were not individually significant but significant in combination with other metabolites (group effect), only metabolites with significant and differential intra-subject temporal change in thrombotic MI versus non-thrombotic MI or stable CAD at an FDR less than 10% were utilized in all multivariate analyses. Such analyses allow for broader characterization of the metabolic changes associated with thrombotic MI within a systems framework. Significance testing of the models produced from these metabolites is independent of the FDR less than 10% filtering step. Metabolites that demonstrated distinct change from acute (T0, T6) to quiescent state between any of the study groups were visualized in radar plots. Partial least squares-discriminant analyses (PLS-DA)[19] utilizing the observed intra-subject temporal changes were conducted to identify biochemical families demonstrating similar patterns of change, and to determine the contributions of families of related metabolites to discriminating the study groups. Two-dimensional 90% confidence interval ellipses were presented in figures of PLS-DA scores utilizing computed Mahalanobis distances to group centroids.[20]

To evaluate the diagnostic utility of the metabolites identified, a statistical classifier was constructed utilizing subject-level baseline abundance values of the metabolites identified in the fold change analysis at time point T0. The goal of constructing a statistical classifier was to determine if the set of metabolites identified as having unique change between acute (T0) and quiescent, or a reduced subset, could be used to discriminate thrombotic MI from non-thrombotic MI and stable CAD at the time of presentation. Given that a subject’s quiescent phase metabolite abundance would not be known at the time of evaluation for suspected thrombotic MI, only the abundance of metabolites at the time of enrollment (T0) were used in the construction of the classifier. Area under the receiver operating curve for discriminating thrombotic MI versus either non-thrombotic MI or stable CAD (AUC) was used to evaluate the performance of the final classifier.[21] Since an independent validation cohort was not available, 10-fold cross-validation was used to estimate the AUC expected in an independent validation cohort.[22] Estimation of the performance of the classifier model was conducted in order to determine if the classifier deserves further validation. To determine the optimal subset of the 65 initial metabolites identified as different between any of the three study groups (thrombotic MI, non-thrombotic MI, stable CAD), the cross-validation estimated misclassification rate was used as a criteria to recursively eliminate half of the metabolites with the lowest relative variable importance.[22] Random Forest (RF) classifiers were employed for classification.[22–24] Additional detail is provided in the supplement (S1 File).

## Results

Eighty subjects were enrolled in this study. To ensure accurate assignment of subjects to study groups, subjects with indeterminate diagnosis were eliminated from the analysis. A total of 38 subjects: 11 acute thrombotic MI, 12 acute non-thrombotic MI, and 15 stable CAD subjects met the stringent analysis criteria (Table 1). Of these, 9 subjects in each MI group and 14 stable CAD subjects had both acute and quiescent state samples for inclusion in the acute change analyses (S1 Fig). The median quiescent state follow-up time was 3.2 months after enrollment. Subjects served as their own control for evaluation of metabolites that changed at the time of acute MI (time of cardiac catheterization for stable CAD subjects) and quiescent state follow-up. Significant differences between study groups in the pattern of intra-subject medication change from time of enrollment (T0) to quiescent state follow-up were limited to heparin (Table 2). However, the percentage of subjects (77.8%) receiving heparin at time of enrollment (T0) was identical between thrombotic and non-thrombotic MI subjects, and the pattern of change between enrollment and follow-up in heparin was also the same for thrombotic and non-thrombotic MI subjects and therefore should not explain metabolic differences observed between these two groups. Excluding any constrained differences resulting from the enrollment criteria, the non-thrombotic MI group had fewer Caucasians, more subjects with a history of stroke, higher heart rate at time of presentation, longer time between presentation and T0, and lower baseline glucose as compared to subjects with thrombotic MI and stable CAD subjects (Table 3).

**Table 2:** Medication use at the acute state (T0, enrollment) versus the quiescent state (follow-up) within and between groups.

Medication	Group	Medications Prior to Enrollment^†^ n(%)	Medications At Follow-Up Visit n(%)	p-value^‡^
Aspirin	Thrombotic MI	9 (100.0)	9 (100.0)	1.00
	Non-Thrombotic MI	9 (100.0)	7 (77.8)	0.47
	Stable CAD	14 (100.0)	14 (100.0)	1.00
				0.15*
P2Y12 Inhibitors	Thrombotic MI	7 (77.8)	9 (100.0)	0.47
	Non-Thrombotic MI	5 (55.6)	3 (33.3)	0.64
	Stable CAD	11 (78.6)	9 (64.3)	0.68
				1.00*
Warfarin	Thrombotic MI	0 (0.0)	0 (0.0)	1.00
	Non-Thrombotic MI	1 (11.1)	3 (33.3)	0.58
	Stable CAD	0 (0.0)	0 (0.0)	1.00
				0.15*
Heparin	Thrombotic MI	7 (77.8)	0 (0.0)	**0.002**
	Non-Thrombotic MI	7 (77.8)	0 (0.0)	**0.002**
	Stable CAD	1 (7.1)	0 (0.0)	1.00
				0.0002*
Anti-Thrombin	Thrombotic MI	2 (22.2)	0 (0.0)	0.47
	Non-Thrombotic MI	0 (0.0)	0 (0.0)	1.00
	Stable CAD	0 (0.0)	0 (0.0)	1.00
				0.15*
Glycoprotein IIb/IIIa Inhibitors	Thrombotic MI	1 (11.1)	0 (0.0)	1.00
	Non-Thrombotic MI	0 (0.0)	0 (0.0)	1.00
	Stable CAD	0 (0.0)	0 (0.0)	1.00
				0.56*
Statins	Thrombotic MI	4 (44.4)	9 (100.0)	**0.03**
	Non-Thrombotic MI	2 (22.2)	5 (55.6)	0.33
	Stable CAD	12 (85.7)	11 (78.6)	1.00
				0.30*
ACE Inhibitors/ARBs	Thrombotic MI	3 (33.3)	4 (44.4)	1.00
	Non-Thrombotic MI	5 (55.6)	5 (55.6)	1.00
	Stable CAD	9 (64.3)	7 (50.0)	0.70
				0.67*
Beta Blockers	Thrombotic MI	3 (33.3)	7 (87.5)	0.15
	Non-Thrombotic MI	2 (22.2)	7 (87.5)	0.06
	Stable CAD	10 (71.4)	10 (71.4)	1.00
				0.11*
Steroids	Thrombotic MI	1 (11.1)	0 (0.0)	1.00
	Non-Thrombotic MI	1 (11.1)	1 (11.1)	1.00
	Stable CAD	0 (0.0)	0 (0.0)	1.00
				0.17*
NSAIDs (Excluding Aspirin)	Thrombotic MI	4 (44.4)	2 (22.2)	0.62
	Non-Thrombotic MI	2 (22.2)	1 (12.5)	1.00
	Stable CAD	0 (0.0)	3 (23.1)	0.22
				0.88*
Pressors	Thrombotic MI	1 (11.1)	0 (0.0)	1.00
	Non-Thrombotic MI	1 (11.1)	0 (0.0)	1.00
	Stable CAD	0 (0.0)	0 (0.0)	1.00
				0.31*
Blood Products	Thrombotic MI	(0.0)	(0.0)	1.00
	Non-Thrombotic MI	(0.0)	(0.0)	1.00
	Stable CAD	(0.0)	(0.0)	1.00
				1.00*

^†^Chronic home medication or within 24 hours preceding enrollment (prior to T0 sample collection) except for warfarin, statins, ACE Inhibitors / ARBs and beta blockers which are home use only.

^‡^p-value for difference in distribution of medication within group between enrollment and follow up

*p-value for difference in distribution of temporal medication changes across study groups

ACE = angiotensin converting enzyme, ARB = angiotensin receptor blocker, NSAID = non-steroidal anti-inflammatory drugs

**Table 3:** Baseline subject characteristics.

Variable	Acute thrombotic MI (N = 9)	Non-thrombotic MI (N = 9)	Stable CAD (N = 14)	p-value
Age (mean ± SD) yrs	57.2±14.7	57.1±17.3	64.5±9.2	0.66
Males (%)	66.7	55.6	57.1	1.00
Caucasian race (%)	100.0	66.7	92.9	**0.03**
Current smoker (%)	44.4	44.4	14.3	0.19
History of dyslipidemia (%)	44.4	22.2	85.7	0.62
History of diabetes mellitus (%)	22.2	11.1	42.9	0.29
History of hypertension (%)	66.7	88.9	92.9	0.34
History of atherosclerosis (%) (MI, CAD, PCI, CABG)	22.2	33.3	100.0	<0.0001
History of congestive heart failure (%)	0.0	0.0	7.1	1.00
History of chronic renal failure (%)	0.0	11.1	0.0	1.00
History of stroke (%)	0.0	33.3	0.0	**0.03**
HR at time of presentation (mean ± SD)	82.8±9.7	91.2±26.3	66.3±9.8	0.002*
MAP at time of presentation (mean ± SD)	104.4±24.7	105.4±21.6	92.0±14.6	0.20
BMI at time of presentation (mean ± SD)	29.2±8.0	28.0±7.5	33.8±6.6	0.14
Time (hours) from presentation to T0 (median ± IQR)	1.38±0.83	19.2±10.7	NA	**0.0006**^†^
Time (hours) symptoms to T0 (median ± IQR)	8.4±20.8	24.3±23.0	NA	0.08^†^
Percentage subjects with peak troponin ≥ 0.12 ng/mL (Ortho Vitros assay) or ≥ 0.5 ng/mL (Beckman assay)	100.0	100.0	0.0	<0.0001
Glucose at baseline (mean ± SD, range)	154.0±38.6, 123.0	106.4±26.5, 83.0	133.1±31.2	**0.01**^†^
Creatinine at baseline (mean ± SD, range)	1.03±0.47, 1.60	0.96±0.39, 1.10	0.92±0.18	0.87^†^
Platelets at baseline (mean ± SD, range)	186.5±84.2, 240.3	217.3±54.1, 156.0	235.8±56.6	0.31*
ST elevation on EKG at baseline (%)	88.9	22.2	0.0	<0.0001
At least one vessel with ≥50% coronary stenosis on enrollment angiogram (%)	100.0	33.3	64.3	0.01
PCI at time of enrollment (%)	100.0	0.0	14.3	**<0.0001**
Aspirin use at time of enrollment (%)	100.0	100.0	85.7	0.49
P2Y12 inhibitors use at enrollment (%)	77.8	55.6	57.1	0.65

*Welch's ANOVA.

^†^Kruskal-Wallis test.

MI = myocardial infarction, CAD = coronary artery disease, PCI = percutaneous coronary intervention, CABG = coronary artery bypass grafting, HR = heart rate, SBP = systolic blood pressure, DBP = diastolic blood pressure, MAP = mean arterial pressure, BMI = body mass index, ACE-I = angiotensin converting enzyme inhibitor

An overview of the analytical approach is summarized in Fig 1. A total of 1,032 metabolites (656 known and 376 unnamed) were quantified per plasma sample. Median relative standard deviation was 4% for the internal standards (instrument variability) and 9% endogenous metabolites (total process variability).

### Patterns of metabolic change

We executed a unique study design that leveraged specific outcomes (as opposed to heterogeneous or composite outcomes), used subjects as their own controls from time of acute disease to quiescent state (as opposed to comparing between subject groups alone), and selected controls by matching on the underlying disease (atherosclerosis) rather than an arbitrarily selected set of risk factors for the targeted disease state (e.g., hypertension). This was accomplished by developing and applying stringent criteria to include only subjects with histologically confirmed thrombotic (type 1) MI, and comparing changes (time of acute MI versus quiescent state) in this group of subjects to change over the same time course in two unique control groups undergoing cardiac catheterization, non-thrombotic MI and stable coronary artery disease (CAD). The non-thrombotic MI group was employed to control for non-specific acute disease processes and ischemia / necrotic change specifically. Non-thrombotic MI was defined via novel criteria that included stringent troponin and blinded angiographic coronary flow / tissue perfusion analysis criteria (Table 1). The stable CAD group was utilized to control for metabolic changes secondary to the underlying disease state, atherosclerosis. Both control groups underwent cardiac catheterization, which provided control for changes secondary to this diagnostic / therapeutic intervention.

**Fig 2:**
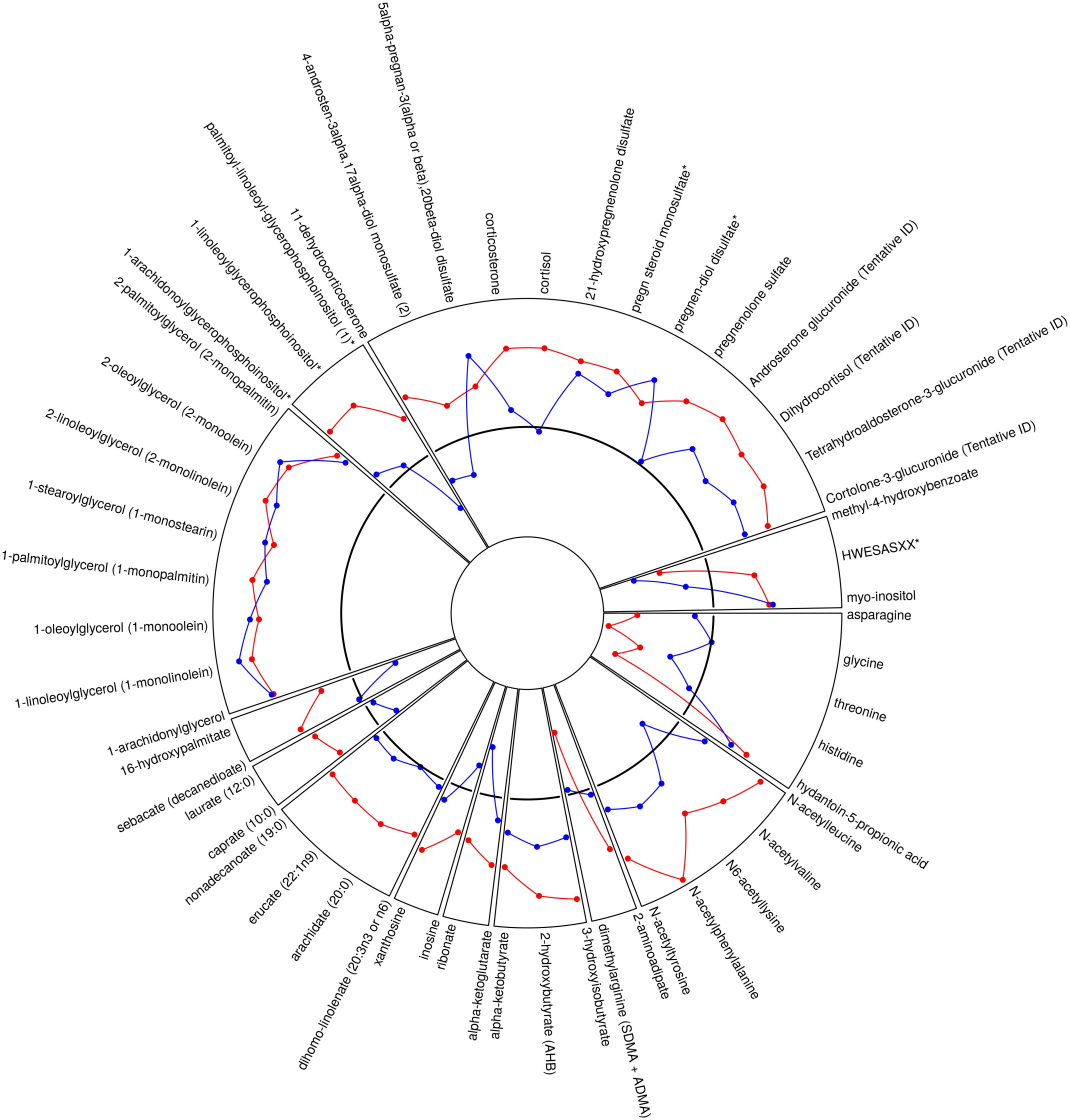
Radar plot of all identified metabolites that demonstrated a significant mean intra-subject temporal change between acute (T0, enrollment prior to cardiac catheterization) and quiescent state in thrombotic MI that is distinct from the pattern of change observed in non-thrombotic MI or stable CAD. The black solid line represents change in stable CAD subjects, red illustrates change in thrombotic MI, and blue illustrates change in non-thrombotic MI. Values above indicate positive change and values below indicate negative change relative to change observed in stable CAD. The radar plot shows 65 metabolites.

**Fig 3:**
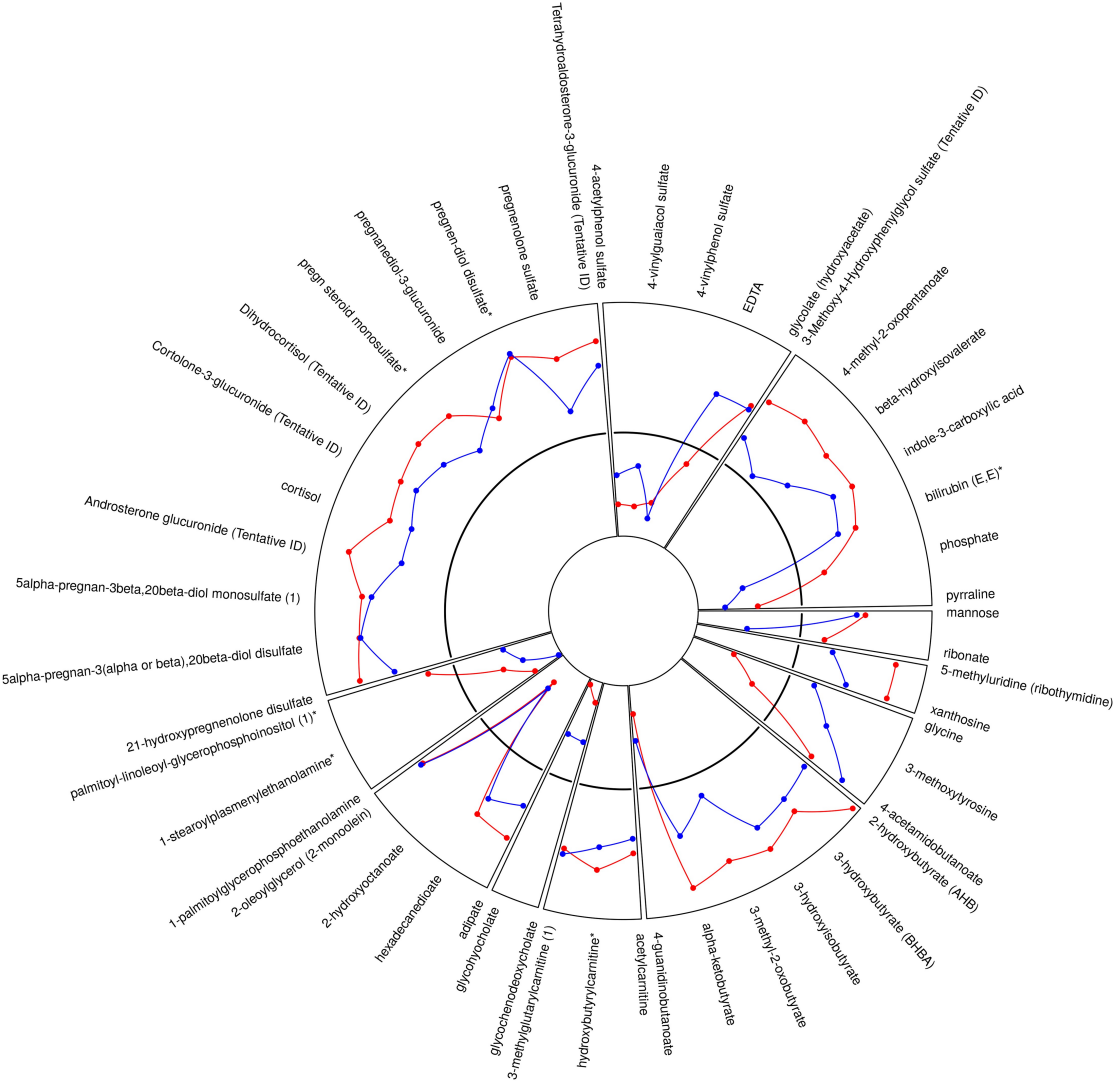
Radar plot of all identified metabolites that demonstrated a significant mean intra-subject temporal change between acute (T6, after cardiac catheterization) and quiescent state in thrombotic MI that is distinct from the pattern of change observed in non-thrombotic MI or stable CAD. The black solid line represents change in stable CAD subjects, red illustrates change in thrombotic MI, and blue illustrates change in non-thrombotic MI. Values above indicate positive change and values below indicate negative change relative to change observed in stable CAD. The radar plot shows 79 metabolites.

**Fig 4:**
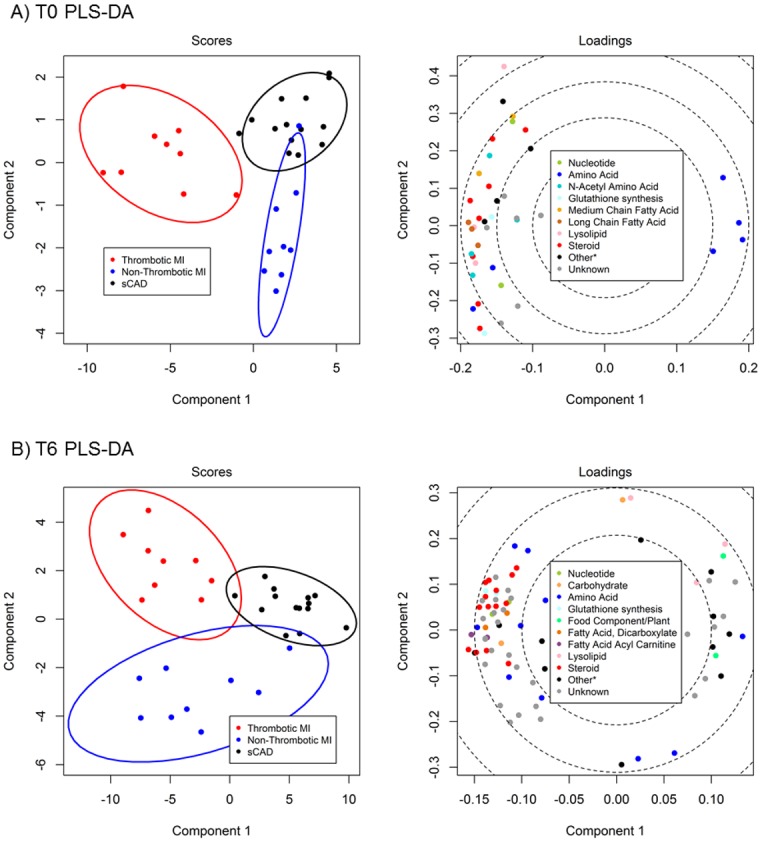
Partial Least Squares-Discriminant Analyses (PLS-DA) of acute over quiescent intra-subject fold changes. Analyses were restricted to metabolites with significant mean intra-subject fold change in acute thrombotic MI that differed between groups at q<0.10. Loadings plots suggest the contributions of biochemical families of related metabolites to discriminating the study groups based on intra-subject fold change from quiescent to acute phase. **(A)** PLS-DA of enrollment (T0) over quiescent intra-subject fold changes. **(B)** PLS-DA of T6 (6 hours post enrollment) over quiescent intra-subject fold changes.

We identified metabolites that changed significantly from acute to quiescent state in thrombotic MI subjects but did not change in the other study groups over the same time course. This mitigated the confounding effects of non-specific acute disease, myocardial ischemia / necrosis, therapeutics, and procedures. For example, lidocaine was identified as a metabolite with significant intra-subject change between the acute and quiescent states; however, a similar change was observed in all three study groups, and was therefore eliminated as a metabolite related to acute thrombotic MI (S2 Fig). Sixty-five metabolites demonstrated a significant (FDR <10%) mean intra-subject change between the acute (T0) and quiescent state (>3 mo follow-up) in thrombotic MI subjects, in which the observed change differed across study groups (Figs 1 and 2). Seventy-nine metabolites were different between T6 and quiescent state (Figs 1 and 3) and differed across study groups. The Partial Least Squares-Discriminant Analyses (PLS-DA) of metabolic change at the time of presentation (T0) relative to quiescent and T6 relative to quiescent produced a metabolic signature that was predictive of each study group (thrombotic-MI, non-thrombotic MI, stable CAD) (*R*^2^*Y* = 0.80 at T0 and *R*^2^*Y* = 0.77 at T6) (Fig 4). Two-dimensional 90% confidence interval ellipses derived from the PLS-DA scores demonstrated a significant degree of separation between thrombotic MI, non-thrombotic MI, and stable CAD at both acute time points (T0 & T6) by metabolic characteristics. The T0 PLS-DA model demonstrates that a temporal increase (T0 relative to quiescent) in certain steroid hormones, lysophospholipids, monoacylglycerols, and n-acetyl amino acids accompanied by a temporal decrease in certain plasma amino acids distinguish thrombotic MI from non-thrombotic MI and stable CAD subjects. While less significant trends by biochemical family emerged in the analysis of T6 PLS-DA model component loadings, a temporal increase (T6 relative to quiescent) in certain steroid hormones remained a characteristic feature distinguishing thrombotic MI from non-thrombotic MI and stable CAD subjects (Fig 4).

From the set of 65 metabolites that demonstrated significant (FDR <10%) mean intra-subject change between the acute (T0) and quiescent state (>3 mo follow-up) in thrombotic MI subjects, in which the change was different across groups, a statistical classifier was constructed. Given that a subject’s quiescent phase metabolite abundance would not be known at the time of evaluation for suspected thrombotic MI, metabolite abundances at the time of enrollment only were utilized from all 11 thrombotic MI, 12 non-thrombotic, and 15 stable CAD subjects to construct a classifier. Ten-fold cross-validation was used to estimate the performance of the final classifier composed of 17 metabolites (Table 4). Cross validation-estimated AUC for the final Random Forest classifier was 0.94 with an estimated sensitivity of 100% and an estimated specificity of 93% for classifying thrombotic MI as compared to both control groups (non-thrombotic MI and stable CAD).

**Table 4:** Seventeen metabolites included in the final Random Forest classifier.

Biochemical	Super	Sub	Platform	RI	Mass	Relative Importance
Androsterone glucuronide (Tentative ID)	Lipid	Steroid	LC/MS Neg	4151	525.2706	44.06
pregn steroid monosulfate*	Lipid	Steroid	LC/MS Neg	5000	397.2054	38.51
pregnenolone sulfate	Lipid	Steroid	LC/MS Neg	5100	395.1898	35.94
Tetrahydroaldosterone-3-glucuronide (Tentative ID)	Lipid	Steroid	LC/MS Neg	4678	541.2647	33.50
cortisol	Lipid	Steroid	LC/MS Pos	4561.9	363.2166	32.57
Dihydrocortisol (Tentative ID)	Lipid	Steroid	LC/MS Pos	4402	365.2315	32.49
Unknown 167			LC/MS Neg	3800	211.0247	32.36
2-linoleoylglycerol (2-monolinolein)	Lipid	Monoacylglycerol	LC/MS Neg	6250	279.2329	32.23
2-oleoylglycerol (2-monoolein)	Lipid	Monoacylglycerol	LC/MS Neg	6950	281.2486	31.16
2-palmitoylglycerol (2-monopalmitin)	Lipid	Monoacylglycerol	LC/MS Neg	6628.9	255.2329	30.61
corticosterone	Lipid	Steroid	LC/MS Pos	4851.2	347.2217	30.13
1-palmitoylglycerol (1-monopalmitin)	Lipid	Monoacylglycerol	LC/MS Neg	6400	255.2329	29.96
Cortolone-3-glucuronide (Tentative ID)	Lipid	Steroid	LC/MS Pos	4858	560.306	29.76
histidine	Amino Acid	Histidine Metabolism	LC/MS Neg	755.9	154.0622	26.97
1-linoleoylglycerol (1-monolinolein)	Lipid	Monoacylglycerol	LC/MS Neg	6477	279.2329	25.39
1-oleoylglycerol (1-monoolein)	Lipid	Monoacylglycerol	LC/MS Neg	6794	281.2486	23.32
1-arachidonylglycerol	Lipid	Monoacylglycerol	LC/MS Neg	6450	303.2329	22.53

### Individual significant candidates

To identify changes in metabolites specific to thrombotic MI as compared with both non-thrombotic MI and stable CAD, two criteria were imposed. First, significant dynamic change in a metabolite at the time of acute thrombotic MI (quiescent versus acute phase) was required, using each subject’s quiescent abundance as a baseline. Second, to ensure that the observed intra-subject changes from a stable disease state to acute state were specific to thrombotic MI, these changes were contrasted to those observed in subjects undergoing the same procedure (cardiac catheterization) with acute non-thrombotic MI (ischemia and non-specific acute illness control) and stable CAD subjects with the same underlying disease state (atherosclerosis).

**Fig 5:**
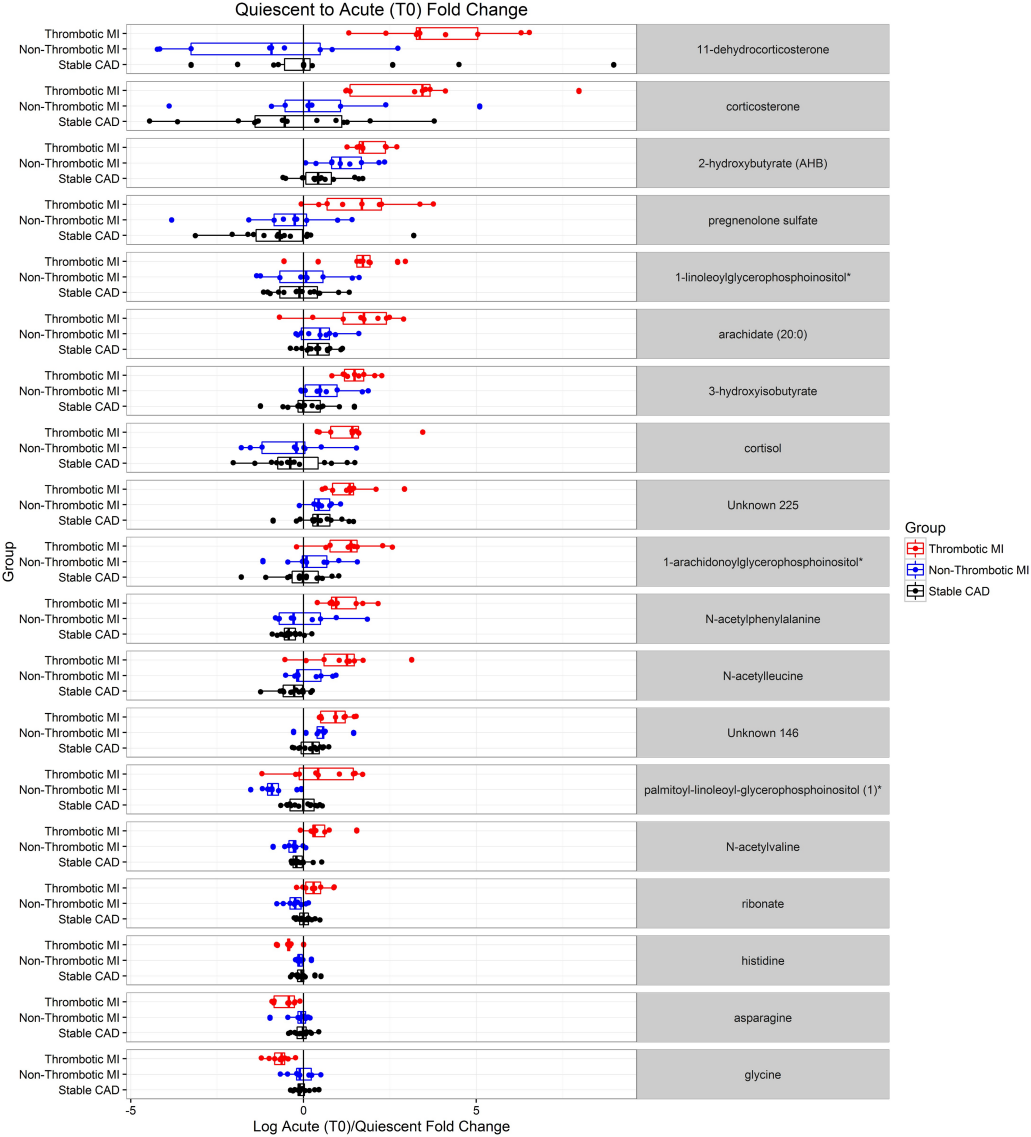
Dot plots illustrating subject level acute (enrollment, T0) over quiescent state fold change for metabolites that demonstrated intra-subject change in thrombotic MI that differed from non-thrombotic MI and sCAD controls at q<0.05.

**Fig 6:**
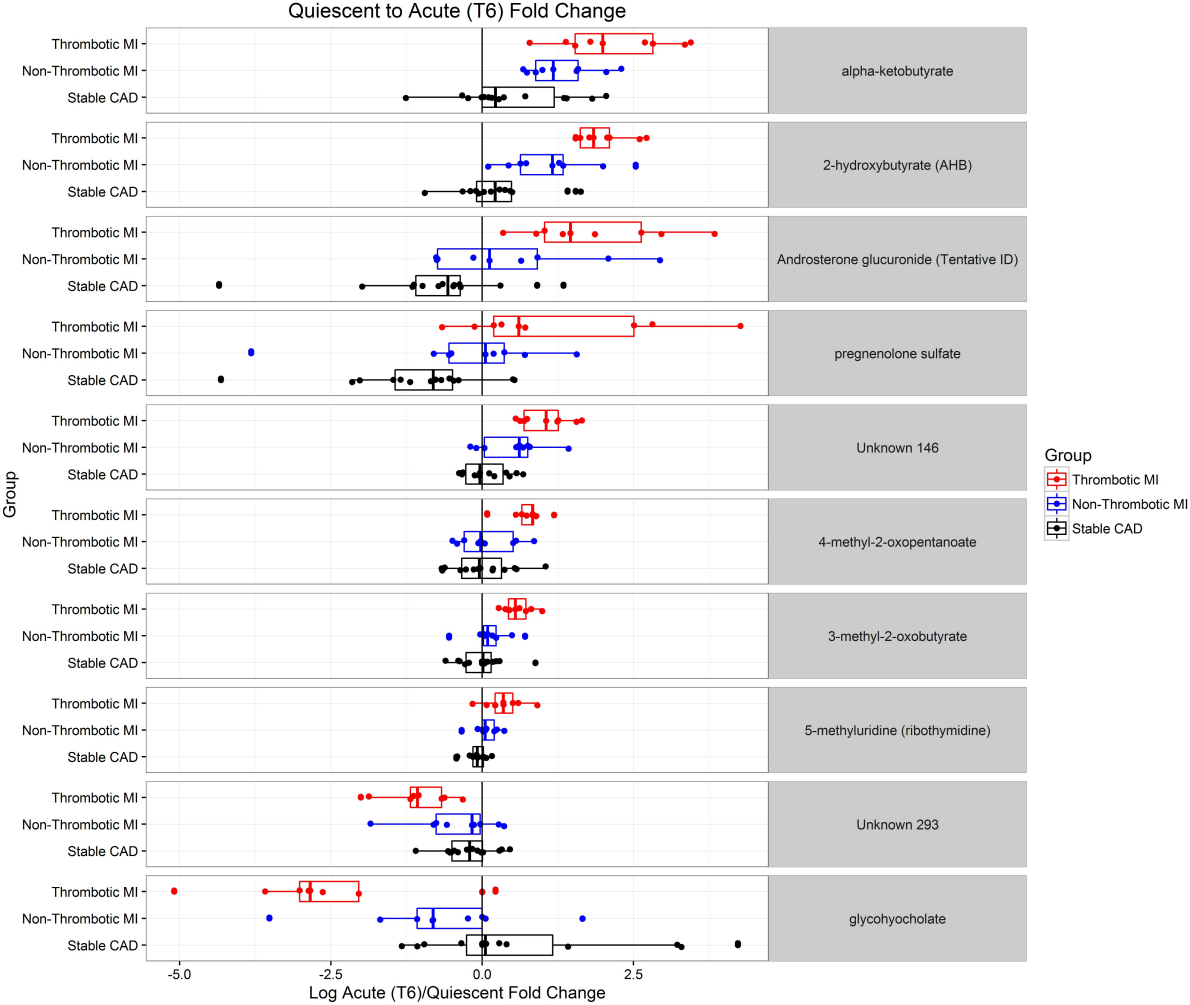
Dot plots illustrating subject level acute (post cardiac catheterization, T6) over quiescent state fold change for metabolites that demonstrated intra-subject change in thrombotic MI that differed from non-thrombotic MI and sCAD controls at q<0.05.

Nineteen metabolites demonstrated a significant mean intra-subject change (FDR <5%) between acute (T0) and quiescent state in thrombotic MI subjects, in which the change was significantly different (FDR <5%) from changed observed in non-thrombotic MI and stable CAD subjects over the same time course (Table 5 and Fig 5). These 19 distinct metabolites consist mainly of lipids (steroid hormones and lysophospholipids in particular), 2-hydroxybutyrate, and amino acids including histidine, glycine, asparagine, n-acetylphenylalanine, n-acetylleucine, and n-acetylvaline. Ten metabolites demonstrated a significant mean intra-subject change (FDR <5%) between acute (T6) and quiescent state in thrombotic MI subjects, in which the change was significantly different (FDR <5%) from change observed in non-thrombotic MI and stable subjects over the same time course (Table 6 and Fig 6). These 10 distinct metabolites consist mainly of lipids, steroid hormones in particular, and 2-hydroxybutyrate. Pregnenolone sulfate, 2-hydroxybutyrate, and an unnamed metabolite demonstrated significantly different mean intra-subject changes between thrombotic MI and both non-thrombotic MI and stable CAD subjects at both acute time points (T0 or T6) and the quiescent state.

**Table 5:** Metabolites specific to thrombotic MI evidenced by T0/Q intra subject fold change. Metabolites with an ANOVA *q* < 0.05 (preserving the false discovery rate at < 5%), significant post-hoc comparisons between thrombotic MI and both control groups, and demonstrating significant change from quiescent to acute (*q* < 0.05) are deemed to be specific to thrombotic MI.

					*Fold Change (T0/Q)*			*ANOVA*		*Post-Hoc Tests*		
Biochemical	Family	Platform	RI	Mass	Thromb. MI	Non-Thromb. MI	Stable CAD	p-value	q-value	Thromb. vs Non-Thromb.	Thromb. vs sCAD	Non-Thromb. vs sCAD
N-acetylphenylalanine	Amino Acid	LC/MS Neg	2597	206.0823	2.18	1.05	0.76	0.00001	0.00306	0.00072	0.00000	0.07661
Glycine	Amino Acid	GC/MS	1486.9	218.1	0.62	0.96	0.96	0.00003	0.00446	0.00007	0.00002	0.98482
N-acetylvaline	Amino Acid	LC/MS Neg	1704	158.0823	1.39	0.81	0.92	0.00006	0.00650	0.00003	0.00024	0.20156
3-hydroxyisobutyrate	Amino Acid	LC/MS Polar	1619.1	103.0401	2.84	1.58	1.08	0.00009	0.00790	0.00928	0.00002	0.05258
N-acetylleucine	Amino Acid	LC/MS Neg	2400	172.0979	2.17	1.11	0.81	0.00012	0.00915	0.00471	0.00003	0.11955
2-hydroxybutyrate (AHB)	Amino Acid	GC/MS	1169.4	131	3.69	2.27	1.42	0.00022	0.01048	0.03699	0.00005	0.02674
Palmitoyl-linoleoyl-glycerophosphoinositol (1)*	Lipid	LC/MS Polar	910	833.5185	1.46	0.56	0.98	0.00025	0.01048	0.00006	0.03846	0.00515
Histidine	Amino Acid	LC/MS Neg	755.9	154.0622	0.73	0.94	0.96	0.00034	0.01192	0.00110	0.00014	0.69936
1-linoleoylglycerophosphoinositol*	Lipid	LC/MS Neg	5494	595.2889	3.00	1.03	0.94	0.00062	0.01825	0.00163	0.00027	0.75354
11-dehydrocorticosterone	Lipid	LC/MS Pos	4533.8	345.206	15.52	0.46	1.60	0.00072	0.01992	0.00020	0.00511	0.10644
Cortisol	Lipid	LC/MS Pos	4561.9	363.2166	2.64	0.80	0.85	0.00074	0.01992	0.00085	0.00054	0.82981
Ribonate	Carbohydrate	LC/MS Polar	2425	165.0405	1.26	0.84	1.03	0.00082	0.02130	0.00019	0.02467	0.02553
Unknown 146		LC/MS Pos	1807	207.0175	1.89	1.41	1.15	0.00123	0.02862	0.03483	0.00028	0.10317
Pregnenolone sulfate	Lipid	LC/MS Neg	5100	395.1898	3.29	0.69	0.64	0.00123	0.02862	0.00226	0.00058	0.87066
1-arachidonoylglycerophosphoinositol*	Lipid	LC/MS Neg	5482	619.2889	2.47	1.20	0.96	0.00137	0.02974	0.00872	0.00037	0.36496
Corticosterone	Lipid	LC/MS Pos	4851.2	347.2217	9.88	1.33	0.75	0.00195	0.03608	0.01037	0.00055	0.39706
Unknown 225		LC/MS Pos	1962	250.093	2.61	1.42	1.37	0.00234	0.03854	0.00409	0.00105	0.84077
Asparagine	Amino Acid	LC/MS Polar	2951.1	131.0462	0.70	0.90	0.98	0.00295	0.04323	0.01588	0.00081	0.36888
Arachidate (20:0)	Lipid	LC/MS Neg	6295	311.2956	2.95	1.37	1.35	0.00330	0.04733	0.00438	0.00167	0.95597

**Table 6:** Metabolites specific to thrombotic MI evidenced by T6/Q intra-subject fold change. Metabolites with an ANOVA *q* < 0.05 (preserving the false discovery rate at < 5%), significant post-hoc comparisons between thrombotic MI and both control groups, and demonstrating significant change from quiescent to acute (*q* < 0.05) are deemed to be specific to thrombotic MI.

					*Fold Change (T6/Q)*			*ANOVA*		*Post-Hoc Tests*		
Biochemical	Family	Platform	RI	Mass	Thromb. MI	Non-Thromb. MI	Stable CAD	p-value	q-value	Thromb. vs Non-Thromb.	Thromb. vs sCAD	Non-Thromb. vs sCAD
2-hydroxybutyrate (AHB)	Amino Acid	GC/MS	1169.4	131	3.94	2.19	1.26	0.00002	0.00657	0.01344	0.00000	0.01076
Unknown 146		LC/MS Pos	1807	207.01747	2.06	1.42	1.04	0.00003	0.00657	0.01133	0.00001	0.01771
Alpha-ketobutyrate	Amino Acid	LC/MS Polar	940	101.02442	4.59	2.51	1.37	0.00016	0.01202	0.03589	0.00004	0.02128
Androsterone glucuronide (Tentative ID)	Lipid	LC/MS Neg	4151	525.27057	3.52	1.39	0.61	0.00030	0.01607	0.03449	0.00007	0.03640
5-methyluridine (ribothymidine)	Nucleotide	LC/MS Pos	1829.3	259.09247	1.27	1.04	0.94	0.00046	0.01607	0.01304	0.00010	0.12510
glycohyocholate	Lipid	LC/MS Neg	5020	464.30176	0.19	0.61	1.58	0.00055	0.01736	0.03390	0.00012	0.05844
3-methyl-2-oxobutyrate	Amino Acid	LC/MS Neg	1465	115.04006	1.50	1.09	0.99	0.00083	0.02075	0.00678	0.00022	0.32426
4-methyl-2-oxopentanoate	Amino Acid	LC/MS Neg	2170	129.05571	1.66	1.05	1.00	0.00177	0.03217	0.00421	0.00069	0.71374
Unknown 293		LC/MS Pos	1037	203.13875	0.47	0.75	0.86	0.00209	0.03364	0.01081	0.00059	0.40231
Pregnenolone sulfate	Lipid	LC/MS Neg	5100	395.18976	2.27	0.81	0.47	0.00342	0.04558	0.03408	0.00084	0.21458

## Discussion

This study identifies 19 metabolites with an intra-subject fold change from time of acute thrombotic MI presentation to the quiescent state, all of which are distinct from any change measured in both the non-thrombotic MI and stable CAD groups undergoing cardiac catheterization over the same time course. Therefore, the changes observed in these metabolites at the time of acute thrombotic MI are unlikely to be related to the cardiac catheterization procedure (performed in both control groups), non-specific stress of acute illness or myocardial necrosis (present in the non-thrombotic MI control), and by association are likely related to plaque disruption and / or coronary thrombosis resulting in acute MI. The unique metabolic changes associated with the acute phase of thrombotic MI, as compared to acute phase of non-thrombotic MI and stable CAD, consist mainly of lipids, steroid hormones (lysophospholipids in particular), 2-hydroxybutyrate, and amino acids including histidine, glycine, asparagine, n-acetylphenylalanine, n-acetylleucine, and n-acetylvaline. These findings warrant further investigation given the potential for leading to new biological understanding of the mechanisms underlying plaque disruption, resultant coronary thrombosis, ischemia, and the development of novel therapies for one of the world’s most common cause of death—acute MI.

A candidate diagnostic model was produced from metabolites that demonstrated an intra-subject fold change from the time of acute MI (cardiac catheterization) to a quiescent state that differed between thrombotic MI, non-thrombotic MI, and stable CAD subjects over the same time course. The resultant 17 metabolite model consisted of multiple lipids (steroids in particular) and histidine, and it performed well via confusion matrix estimates for the differentiation of thrombotic from non-thrombotic MI and stable CAD, utilizing metabolite abundance at presentation alone. These metabolites hold promise and warrant further validation for more efficient diagnosis of acute thrombotic MI, prior to irreversible myocardial necrosis, and for the ability to differentiate thrombotic MI from non-thrombotic MI. Additionally, this set of metabolites may be representative of common biological processes, e.g., lipid / monoacylglycerol metabolism, that cause or are triggered by thrombotic MI.

National guidelines state that cardiac troponins are the most sensitive and specific biomarkers for non-ST elevation myocardial infarctions[25], but these measures are specific for myocardial necrosis, not for coronary thrombosis. Hence, these measurements cannot distinguish tissue necrosis caused by coronary thrombosis (type 1 MI) from other causes of necrosis such as demand ischemia (type 2 MI) or stress cardiomyopathy.[1] One recent study of all troponin tests ordered by treating physicians in a hospital system found 42% of these tests to be positive; 29% were secondary to a non-thrombotic (type 2) troponin elevation as compared to 13% from an acute MI diagnosis.[3] Mortality was 59% at 3.2 years in the patients with non-thrombotic (type 2) troponin elevations.[3] Troponin is further limited by the fact that it is a measure of myocardial necrosis, the outcome of the triggering event, rather than the cause and therapeutic target itself (i.e., plaque disruption and ensuing thrombosis). Therefore, troponin elevations often fail to confirm the diagnosis of acute MI before the induction of irreversible myocardial necrosis, even with highly sensitive cardiac troponin assays.[5] This study provides data supportive of the hypothesis that modeling of the abundance of candidate metabolites at the time of presentation holds promise for the development of new diagnostic tools that will expeditiously, efficiently, and specifically diagnose acute MI, including differentiating thrombotic MI from non-thrombotic MI.

Although steroid hormones increase in the circulation after an acute emotional or physiological stress,[26, 27] both acute and chronic stress have been established as independent risk factors for acute MI.[28, 29] Circulating steroid hormone levels and the incidence of acute MI both peak shortly after rising from sleep.[26, 27, 30, 31] Similarly, we find that the abundance of multiple steroid hormones and amino acids in plasma are different at the time of thrombotic MI compared with the quiescent state in the same patients; significant changes in these metabolites were not observed in plasma from non-thrombotic MI and stable CAD subjects. These findings suggest that changes in stress hormones may be related to factors other than the acute stress response (e.g., thrombosis) or may indicate that patterns of circulating stress hormones may differ by the inciting underlying “stressor.” Previously, it was found that cortisol levels are 70% sensitive and 85% specific in differentiating acute MI from angina among patients admitted to an intensive care unit.[32] A simple cortisol / norepinephrine ratio has been shown to differentiate posttraumatic stress disorder from depression, bipolar disorder, and schizophrenia at the time of hospital admission.[33] Copeptin, secreted as part of the hypothalamic stress response, has been shown to aid in the diagnosis of acute MI in a multicenter study of 1,967 patients presenting to the emergency room with chest pain.[34]

It is possible that the metabolites found to be different in plasma from thrombotic MI patients may contribute to the thrombotic response. Steroid hormones have been associated with a hypercoagulable state characterized by increased platelet activity, reduced fibrinolytic capacity, increased heart rate and blood pressure—all possible mechanistic links between stress, steroid hormones, and acute MI.[31, 35–39] In addition, histidine, which was significantly lower at the time of acute thrombotic MI (T0), has been shown to reduce platelet aggregation after vascular injury in animal models.[40] In our candidate 17 metabolite model, histidine was important for differentiating thrombotic MI versus non-thrombotic MI and stable CAD. Our finding of higher steroid hormones and lower histidine in the plasma at the time of acute thrombotic MI, but not in the context of non-thrombotic MI, raises the possibility that these metabolites may contribute to a pathological coronary thrombosis milieu in contradistinction to a “controlled” or “healing” thrombotic response to plaque disruption. Additionally, alternative lines of evidence identify corticosteroids as important markers of thrombosis. In experimental models, platelet-activating factor (PAF) has been shown to increase adrenal cortisol secretion.[41] Whether steroid elevation accompanying thrombotic MI is a causative association (e.g., via modulating coagulability) or an adaptive negative feedback mechanism of platelet activation deserves further study.

At the time of acute thrombotic MI, several amino acids were lower, while several N-acetylated amino acids were higher compared with the quiescent state. This change was distinct from the change in these metabolites over the same time course in non-thrombotic and stable CAD subjects. Acetylation of the amino acid after an N-terminal methionine occurs as part of protein catabolism.[42, 43] The amino acid after methionine in fibrinogen is phenylalanine, raising the possiblity that the N-acetylphenylalanine in the plasma of type 1 MI patients is derived from a breakdown of fibrinogen. Additionally, this may be reflective of increased acetyltransferase / deacetylase activity, which is a rate-limiting step in the production of platelet activating factor.[44, 45] Higher serum phenylalanine was significantly associated with future MI in a metabolomics study of over 13,000 subjects [46] and phenylalanine infusion has been shown to limit post MI stunning via hydroxyl scavenging in an animal model.[47] Serum glycine levels have been directly associated with anti-inflammatory effects in animal models.[48] The role of amino acids, n-acetylated amino acids, and n-acetyltransferase activity at the time of acute thrombotic MI awaits further investigation.

Thrombotic acute myocardial infarction (MI) is most commonly caused by thrombi overlying disrupted atherosclerotic plaques. However, even though plaque disruption often precipitates thrombosis, autopsy studies have shown that plaque rupture alone is not sufficient to cause an occlusive coronary thrombus. Nearly 80% of plaque ruptures do not result in occlusive coronary thrombosis, and are healed via a controlled hemostatic response.[9] Moreover, 30–35% of thrombotic MI resulting in death are secondary to plaque erosion (i.e., thrombus formation despite an intact fibrous plaque with no communication between the necrotic core and luminal blood).[10] Hence, plaque rupture is neither a sufficient nor a necessary condition for the formation of an occlusive thrombus. Therefore, we propose that the magnitude of the thrombotic response to plaque destabilization (rupture or erosion) depends upon the thrombotic potential of the blood, which is determined in part by circulating metabolites that moderate thrombosis and consequently the clinical manifestations of atherothrombosis. While we cannot identify tissue sources of circulating metabolites, the association of several plasma lipids (steroid hormones, lysophospholipids) and amino acids with atherothrombosis suggests that these metabolites may be derived from the process of atherothrombosis itself, or they are characteristics of a metabolic state that heightens the propensity for atherothrombosis. Hence, further identification and validation of the factor(s) that drive a pathologic, rather than a homeostatic or subclinical, response to plaque disruption would be of enormous value in the prevention, diagnosis, and treatment of thrombotic MI.

Our study has several strengths. First, our methodology for the classification of subjects into study groups was designed to create “ideal” circumstances for discovering new biology related to acute thrombotic MI connected to atherosclerotic plaque disruption and the systemic milieu necessary for pathological thrombosis rather than myocardial necrosis alone. Our criteria for classification of acute thrombotic and acute non-thrombotic MI expands upon criteria we previously introduced[15, 16] and will facilitate further research on acute thrombotic and acute non-thrombotic MI. Repeat measures within our subjects, including a quiescent phase, limits inter- and intra-subject variance and confounding by identifying changes within patients.[14] Our statistical analysis selected candidate biomarkers that demonstrated a change between the quiescent phase and acute thrombotic phase time points that were distinct from any changes observed in non-thrombotic MI and stable CAD controls also receiving cardiac catheterization over this time course.

Our study has several limitations. Although our analysis identified over 1000 candidate metabolites, present estimates suggest that the human metabolome consists of over 20,000 endogenous metabolites.[49] As the technology progresses, we expect to be able to produce a more complete metabolic profile. Assessment of circulating plasma does not provide information on the tissue of origin for measured metabolites. However, the circulation has the unique advantage of being the repository for metabolites from all tissues and therefore is reflective of the biological state of the entire patient. A larger sample size would be advantageous. However, many larger studies are not able to produce phenotypes with the level of specificity achieved in this smaller study and are therefore limited by heterogeneous or composite endpoints that likely have heterogeneous biology.[50] We mitigated the small sample size by creating exquisitely specific end points, using subjects as their own controls by identifying change from time of acute disease to quiescent state, and compared this change to change in two control groups, receiving similar therapy, over the same time course. Control groups were designed to match the thrombotic MI group by disease state (myocardial necrosis or underlying atherosclerosis) as opposed to just the risk factors for a disease state. Subjects with acute thrombotic MI were all ST elevation myocardial infarctions requiring immediate cardiac catheterization to meet the standard of care. This resulted in a shorter time interval between presentation and T0 in acute thrombotic MI versus non-thrombotic MI control subjects. The higher heart rate and blood glucose levels at the time of presentation in the acute thrombotic MI group may suggest that the acute thrombotic MI subjects were more acutely ill at time of T0 sampling than the non-thrombotic MI group. In addition, while all subjects in this study received a cardiac catheterization and all of the thrombotic MI subjects received percutaneous coronary intervention (PCI), by study inclusion criteria, none of the non-thrombotic MI subjects received PCI and only 14% of stable CAD subjects received PCI. Therefore, biomarkers related to PCI alone may affect the results. A larger sample size would allow for more in-depth analysis of the impact of different factors (e.g., PCI, medications) on metabolites at the time of acute thrombotic MI. While we employed a robust methodology, we are presenting candidate biomarkers in this study which we believe warrant support for validation in larger independent cohorts. This first-of-its-kind association of metabolites with acute thrombotic MI requires additional experimentation to delineate the validity and nature of this relationship.

## Conclusion

We report the discovery of multiple circulating metabolites specific to the acute thrombotic MI state in comparison with acute non-thrombotic MI and stable CAD. Changes in several lipids (steroids in particular) and amino acid metabolites were specific to acute thrombotic MI as compared to non-thrombotic MI and stable CAD states. These metabolite changes and baseline differences in abundance show promise for differentiating thrombotic versus non-thrombotic MI and stable CAD accurately by model testing. This study introduces research criteria for differentiation of thrombotic and non-thrombotic MI and substantiates the value of high-throughput discovery metabolomics to gain a greater understanding of plaque disruption, pathological thrombus formation, and myocardial ischemia. Rigorous validation of the metabolic differences and relative changes identified in this study has the potential to provide a new direction for the discovery of diagnostic and prognostic biomarkers, and may also hold promise for development of novel therapeutics for the prevention and treatment of acute MI.

## Supporting information

S1 File Supplemental methods. (DOCX)

S1 Fig Enrollment and analysis cohorts. (TIF)

S2 Fig Lidocaine box plot. Box plot of metabolite that demonstrates a significant distributional change from acute to quiescent state in thrombotic MI subjects but has a similar pattern of change in non-thrombotic MI and stable CAD subjects. Therefore, such metabolites are likely related to the procedure at time of enrollment (coronary angiography), acute illness or ischemia—as opposed to thrombotic MI.
(TIFF)

## Abbreviations

CAD: coronary artery disease
FDR: false Discovery Rate
GC-MS: gas chromatography mass spectrometry
MI: myocardial infarction
PAF: platelet-activating factor
PCI: percutaneous coronary intervention
PLS-DA: partial least squares-discriminant analyses
UPLC-MS / MS: ultra-performance liquid chromatography tandem mass spectrometry
