# Supplementary material for: Identification of a plasma metabolomic signature of thrombotic myocardial infarction that is distinct from non-thrombotic myocardial infarction and stable coronary artery disease
Source: PLoS One. 2017 Apr 17;12(4):e0175591. doi: 10.1371/journal.pone.0175591 (PMC5393610; doi:10.1371/journal.pone.0175591)
Supplement: S1 File — (DOCX) [file pone.0175591.s001.docx]

**S1 File. Supplemental methods.**

Enrollment Population: Enrollment criteria for both groups required that each patient be >18 years of age and scheduled for coronary angiography within 24 h. Subjects who received fibrinolysis were not eligible. Those enrolled in the suspected acute MI group must have reported ischemic symptoms lasting >10 min, within 24 h of enrollment, and had to meet at least one of the following four criteria: (1) new or presumably new ST-segment depression >0.1 mV; (2) elevated troponins or CK-MB levels within 24 h of enrollment; (3) ≥1 mm ST-segment elevation in ≥2 contiguous electrocardiogram (ECG) leads; or (4) ≥1 mm ST-segment depression in V1 and V2 (posterior wall infarct). Subjects considered for enrollment in the suspected stable CAD group were required to have presented for angiography as an elective procedure. Patients in the suspected stable CAD group were excluded on the basis of any one of the following criteria: (1) hospitalization for acute coronary syndrome (ACS) or clinical instability within 4 weeks prior to planned enrollment; (2) coronary artery bypass graft (CABG) surgery within 1 year prior to planned enrollment; (3) percutaneous coronary intervention (PCI) within 12 weeks prior to planned enrollment; (4) stroke within 12 weeks prior to planned enrollment; (5) presence of unstable angina or symptoms refractory to maximal medical therapy; (6) presence of significant co-morbidities likely to cause death within 2 years; or (7) significant active history of substance abuse within 5 years of enrollment. Subjects were asked to decline enrollment if they would not be able to return to the medical campus for a 3-month stable follow-up.

Study Cohort: The definition of acute thrombotic MI included: (1) the presence of a histologically confirmed (by blinded pathological assessment) coronary thrombus of 0 to 4 days old, (2) 50% to 100% stenosis in the vessel in which thrombus was recovered, (3) absence of a major coronary dissection (4) an elevated troponin and a >30% increase in troponin between T0 and T6 hours (Table 1). The definition of acute non-thrombotic MI included: (1) no histologically confirmed (by blinded pathological assessment) coronary thrombus, (2) no stenosis >50% or any complex or indeterminate ambrose lesions, no identified filling defects, TIMI flow = 3, TIMI myocardial perfusion grade =3 in all coronary vessels via blinded core laboratory angiogram assessment, (3) an elevated troponin and a >30% increase in troponin between T0 and T6 hours (Table 1).These criteria expand upon those previously proposed by our group([1](#_ENREF_1), [2](#_ENREF_2)) and we believe are more specific than any other published criteria for identifying acute atherothrombotic MI.([3-9](#_ENREF_3))

Patients with stable CAD were identified as those presenting for an elective cardiac catheterization with a past medical history of atherosclerosis, as evidenced by CABG, PCI, stroke/transient ischemic attack (TIA), carotid endarterectomy (CEA), peripheral artery bypass procedure, abdominal aortic aneurysm (AAA) repair, or >50% stenosis in one or more coronary vessels on enrollment angiogram (via blinded core laboratory assessment). Additional criteria included normal thrombolysis in myocardial infarction (TIMI) flow and myocardial perfusion grade (MPG) in all vessels as well as pre- and post-procedure cardiac troponin I <99% for a healthy population specific to the assay used (Table 1). Subjects who did not meet either thrombotic MI, non-thrombotic MI or stable CAD criteria were eliminated from the study to limit confounding from misclassification and to produce a cohort appropriate for discovering new metabolites related to acute thrombotic MI. Additionally, subjects without a quiescent state sample were eliminated from the intra subject change analysis (Supp. Fig. 1).

History, Physical Exam, Clinical Laboratory, and ECG Data: All subjects were evaluated by study personnel, and each subject’s history, physical examination results, clinical laboratory data, and ECG data were collected prior to quantification of metabolites. The subject’s medical records were used to aid in the collection of pertinent medical history. A single study physician (APD) read all ECGs in accordance with *a priori* study guidelines. Standard laboratory data (troponin, creatinine, blood cell, and platelet counts) were obtained from the treating hospital clinical laboratory at standardized study time points: at the time of the angiogram (T0) and 6 (T6) unless the subject was discharged from the hospital prior to this time point. Follow-up history, physical exam results, and laboratory data were collected at a single follow-up (TF/U) visit 3 to 12 (median, 3.27) months after the procedure or hospitalization for acute MI, when the subject was in a stable condition.

Biochemical Analyses: Enrollment sample collection via an arterial sheath took place at the time of coronary angiography after a 5-10mL waste draw. Follow-up samples (6 hours and >3 months) were collected from a peripheral vein, preferably a virgin vein, without a tourniquet (maximum pressure of <40 mmHg via blood pressure cuff), using a 21G needle, after >10 ml of clinical blood collection (waste draw), and into a tube containing ethylenediamine tetraacetic acid (EDTA). Sample processing time was rigorously standardized to begin 45 min after phlebotomy and be completed without interruption.

Serum cardiac troponin I concentrations were measured by either of two independent CLIA-approved laboratories, the University of Louisville or KentuckyOne Jewish Hospital. The Ortho Vitros 5600 assay was used to measure cardiac troponin I in subjects receiving treatment at the University of Louisville Hospital. For this assay, the 99% cut-off level for a healthy population was 0.035 ng/ml with a coefficient of variation (CV) <10%. This assay further defined 0.12 ng/mL as the most efficient (more specific) cut-off point for the diagnosis of acute MI. The Beckman Access assay was used to measure cardiac troponin I in subjects receiving treatment at KentuckyOne Jewish Hospital. For this assay, the 99% cut-off level for a healthy population was 0.04 ng/ml, but a CV <10% was not achieved until 0.06 ng/ml. This assay defined 0.5 ng/mL as the most efficient (more specific) cut-off point for the diagnosis of acute MI.

Coronary Angiographic Assessment: Angiograms were examined in a blinded fashion for all subjects by the Johns Hopkins Quantitative Angiographic Core Laboratory. The criteria for identifying and quantifying coronary thrombosis and atherosclerotic burden were jointly developed by the Core lab and the study team from existing published data.([10-17](#_ENREF_10))

Histological Data: At the hospitals of study enrollment, coronary aspiration, with intent to retrieve the culprit coronary thrombosis, was standard of care in ST-segment elevation myocardial infarction (STEMI) patients. Aspiration attempt was left to the discretion of the subject’s treating interventional cardiologist. All samples from aspiration attempts were strained, immediately preserved in formalin, and sent to CVPath Institute, Inc., Gaithersburg, Maryland, for blinded histological evaluation by a pathologist specialized in the analysis of coronary thrombosis.([18](#_ENREF_18), [19](#_ENREF_19))

Metabolomics:

Quantification of relative metabolite abundance in plasma was performed by Metabolon, Inc. Samples were prepared using an automated MicroLab STAR® system (Hamilton Company, Reno, NV). A recovery standard was added prior to the extraction process for quality control purposes. To remove protein, dissociate small molecules bound to protein or trapped in the precipitated protein matrix, and to recover chemically diverse metabolites, proteins were precipitated with methanol under vigorous shaking for 2 min (Glen Mills GenoGrinder 2000, Clifton, NJ) followed by centrifugation. The resulting extract was divided into fractions for analysis.

UPLC-MS/MS was conducted with positive ion mode electrospray ionization, negative ion mode electrospray ionization, and a negative ionization optimized for polar molecule detection. Molecular identification was performed by Metabolon via a standard reference library based on the retention time on chromatograph, molecular weight (m/z), preferred adducts, in-source fragments, and their associated MS/MS spectra. The integrated nature of the platform, curation software and manual review of all peaks identified allows for identification of a large number and broad spectrum of metabolites with a high degree of confidence.([20-22](#_ENREF_20)) After identification of peaks, molecular abundances were quantified using area-under-the-curve. If the molecular abundance of a biochemical could not be quantified for a sample, the minimum value of the remaining samples was imputed. Raw biochemical data was scaled by dividing abundance values by the median for each biochemical.

Several types of controls were analyzed in concert with the experimental samples: a pool of well-characterized human plasma served as a technical replicate throughout the data set; extracted water samples served as process blanks; and a cocktail of QC standards that were carefully chosen not to interfere with the measurement of endogenous compounds were spiked into every analyzed sample, allowed instrument performance monitoring and aided chromatographic alignment. Reproducibility of results has been demonstrated by this laboratory.([23](#_ENREF_23))

Statistical Analysis:

Area under the receiver operating curve (AUC) was defined as the area under the curve traced by varying the decision boundary (predicted class probability) used for the binary classification of a plasma samples in the space spanned by sensitivity and 1-specificity.([24](#_ENREF_24)) Since an independent validation cohort was not available, 10-fold cross-validation was used to estimate the AUC expected in an independent validation cohort.([25](#_ENREF_25)) For each 10-fold cross-validation, the enrollment abundance data was partitioned into 10 folds. After partitioning, a classifier was trained using 9 folds and then evaluated on the remaining fold. This procedure was repeated 10 times with each subject’s sample evaluated once. To determine if a reduced subset could be used for classification, the number of metabolites included was recursively divided by two and rounded to the nearest integer. The metabolites carried forward at each recursion were those with the greatest importance score defined in a later paragraph.

Random Forest (RF) classifiers were employed for classification. Random Forests were chosen given the robustness of this technique for classification given more predictors (metabolites) than replicates,([26](#_ENREF_26)) presence of noisy and non-linear predictors,([27](#_ENREF_27)) and because RF classifiers provide a measure of predictor importance for each metabolite([25](#_ENREF_25))([27](#_ENREF_27))([1](#_ENREF_1)).([25](#_ENREF_25)) A Random Forest classifier is an ensemble of bootstrapped classification trees with a random subspaces constraint enforced in the construction of individual tree branches. An individual tree, denoted $T_{m}$, is constructed as follows.([25](#_ENREF_25)) Each tree begins with a parent node that is iteratively split into child nodes. To determine an optimal binary split at each iteration, a set of regions $\left\{ R_{L}\left( X_{j},s \right), R_{R}\left( X_{j}, s \right) \right\}$ is defined using each metabolite $X_{j}$ and cut-point from the observed abundance range $s$. For each region, the empirical distribution of samples is determined:  $\hat{p}_{R,g}=\frac{1}{N(R)}\sum_{X_{ij\in R}} I(y_{i}=g),$ where $y_{i}$ denotes the true class (thrombotic MI or non-thrombotic MI) of the $i$th plasma sample and $g$ represents an arbitrary class. A predicted classification is then made for each sample as: $\hat{y}_{i}=\arg\max\left\{ \hat{p}_{R,g} \right\}$. A measure of node impurity is then used as an objective criteria to choose $X_{j}$ and $s$ so as to minimize impurity across nodes. The criteria used in our analysis was misclassification error: $1-\frac{1}{n}\sum_{i=1}^{N} I(\hat{y}_{i}=y_{i})$. Parent nodes are recursively split into child nodes until a minimum node size $n_{0}$ is reached. To aggregate classification trees, $T_{m}$, $m=1,2,\ldots,M$ into a Random Forest ensemble classifier, two additional modifications are required in the construction of the trees. First, individual trees were derived using bootstrapped datasets. A bootstrapped dataset is a random sample drawn with replacement from the original abundance data with the same number of samples as the original. We set $M=500$, so 500 bootstrapped datasets were used to construct individual classification trees. Second, at each iteration only a random subset of metabolites were considered in the generation of regions, $R$. This restriction, known as a random subspaces constraint, was imposed to reduce the correlation between individual trees. The ensemble predicted class probabilities associated with a sample were then $\hat{p}_{i,g}=\frac{1}{N\left( T \right)}\sum_{m=1}^{M} I(\hat{y}_{i,m}=g)$, where $\hat{y}_{i,m}$ is the predicted class of the $i$th sample from tree $T_{m}$.

Given that bootstrap aggregation was utilized to develop an ensemble classifier, a natural estimate of metabolite importance was used for metabolite selection.([25](#_ENREF_25)) As each tree was trained using a bootstrap sample, a set of plasma samples was omitted from each classification tree training set. These samples, known as out of bag (OOB) samples were used to estimate the importance of individual metabolites. To determine the importance of a metabolite, $X_{j}$, the misclassification rate $\epsilon_{m}$ for individual tree $T_{m}$ on the OOB samples was computed. Next the metabolite abundances were randomly permuted for the OOB samples and the misclassification rate was computed again as $\epsilon_{m}'$. Metabolite importance was then computed as: $Imp_{j}=\frac{1}{M}\sum_{m=1}^{M} \epsilon_{m}^{'}-\epsilon_{m}$.

Statistical analyses were conducted in R version 3.2.0 (R Core Team, 2015). Metabolites reported as unidentified by Metabolon, Inc. were tentatively identified using the database MZedDB.([28](#_ENREF_28)) In addition to base R, the packages ggplot2([29](#_ENREF_29)), qvalue([30](#_ENREF_30)), multcomp,([31](#_ENREF_31)) randomForests,([32](#_ENREF_32)) and dplyr,([33](#_ENREF_33)) were used. The manuscript is in compliance with the STROBE (& MOOSE) guidelines for observational studies.

CITATIONS

1. DeFilippis AP, Chernyavskiy I, Amraotkar AR, Trainor PJ, Kothari S, Ismail I, et al. Circulating levels of plasminogen and oxidized phospholipids bound to plasminogen distinguish between atherothrombotic and non-atherothrombotic myocardial infarction. Journal of thrombosis and thrombolysis. 2016;42(1):61-76.

2. DeFilippis AP, Oloyede OS, Andrikopoulou E, Saenger AK, Palachuvattil JM, Fasoro YA, et al. Thromboxane A(2) generation, in the absence of platelet COX-1 activity, in patients with and without atherothrombotic myocardial infarction. Circulation journal : official journal of the Japanese Circulation Society. 2013;77(11):2786-92.

3. Ambrose JA, Loures-Vale A, Javed U, Buhari CF, Aftab W. Angiographic correlates in type 1 and 2 MI by the universal definition. JACC Cardiovascular imaging. 2012;5(4):463-4.

4. Javed U, Aftab W, Ambrose JA, Wessel RJ, Mouanoutoua M, Huang G, et al. Frequency of elevated troponin I and diagnosis of acute myocardial infarction. Am J Cardiol. 2009;104(1):9-13.

5. Melberg T, Burman R, Dickstein K. The impact of the 2007 ESC-ACC-AHA-WHF Universal definition on the incidence and classification of acute myocardial infarction: a retrospective cohort study. International journal of cardiology. 2010;139(3):228-33.

6. Morrow DA, Wiviott SD, White HD, Nicolau JC, Bramucci E, Murphy SA, et al. Effect of the novel thienopyridine prasugrel compared with clopidogrel on spontaneous and procedural myocardial infarction in the Trial to Assess Improvement in Therapeutic Outcomes by Optimizing Platelet Inhibition with Prasugrel-Thrombolysis in Myocardial Infarction 38: an application of the classification system from the universal definition of myocardial infarction. Circulation. 2009;119(21):2758-64.

7. Saaby L, Poulsen TS, Diederichsen AC, Hosbond S, Larsen TB, Schmidt H, et al. Mortality rate in type 2 myocardial infarction: observations from an unselected hospital cohort. The American journal of medicine. 2014;127(4):295-302.

8. Saaby L, Poulsen TS, Hosbond S, Larsen TB, Pyndt Diederichsen AC, Hallas J, et al. Classification of myocardial infarction: frequency and features of type 2 myocardial infarction. The American journal of medicine. 2013;126(9):789-97.

9. Stein GY, Herscovici G, Korenfeld R, Matetzky S, Gottlieb S, Alon D, et al. Type-II myocardial infarction--patient characteristics, management and outcomes. PloS one. 2014;9(1):e84285.

10. Ambrose JA, Almeida OD, Sharma SK, Dangas G, Ratner DE. Angiographic evolution of intracoronary thrombus and dissection following percutaneous transluminal coronary angioplasty (the Thrombolysis and Angioplasty in Unstable Angina [TAUSA] trial). The American journal of cardiology. 1997;79(5):559-63.

11. Ambrose JA, Almeida OD, Sharma SK, Torre SR, Marmur JD, Israel DH, et al. Adjunctive thrombolytic therapy during angioplasty for ischemic rest angina. Results of the TAUSA Trial. TAUSA Investigators. Thrombolysis and Angioplasty in Unstable Angina trial. Circulation. 1994;90(1):69-77.

12. Ambrose JA, Israel DH. Angiography in unstable angina. The American journal of cardiology. 1991;68(7):78B-84B.

13. Capone G, Wolf NM, Meyer B, Meister SG. Frequency of intracoronary filling defects by angiography in angina pectoris at rest. The American journal of cardiology. 1985;56(7):403-6.

14. Gibson CM, Cannon CP, Murphy SA, Marble SJ, Barron HV, Braunwald E, et al. Relationship of the TIMI myocardial perfusion grades, flow grades, frame count, and percutaneous coronary intervention to long-term outcomes after thrombolytic administration in acute myocardial infarction. Circulation. 2002;105(16):1909-13.

15. Gibson CM, Cannon CP, Murphy SA, Ryan KA, Mesley R, Marble SJ, et al. Relationship of TIMI myocardial perfusion grade to mortality after administration of thrombolytic drugs. Circulation. 2000;101(2):125-30.

16. Goldstein JA, Demetriou D, Grines CL, Pica M, Shoukfeh M, O'Neill WW. Multiple complex coronary plaques in patients with acute myocardial infarction. The New England journal of medicine. 2000;343(13):915-22.

17. Zack PM, Ischinger T, Aker UT, Dincer B, Kennedy HL. The occurrence of angiographically detected intracoronary thrombus in patients with unstable angina pectoris. American heart journal. 1984;108(6):1408-12.

18. Kramer MC, Rittersma SZ, de Winter RJ, Ladich ER, Fowler DR, Liang YH, et al. Relationship of thrombus healing to underlying plaque morphology in sudden coronary death. Journal of the American College of Cardiology. 2010;55(2):122-32.

19. Kramer MC, van der Wal AC, Koch KT, Ploegmakers JP, van der Schaaf RJ, Henriques JP, et al. Presence of older thrombus is an independent predictor of long-term mortality in patients with ST-elevation myocardial infarction treated with thrombus aspiration during primary percutaneous coronary intervention. Circulation. 2008;118(18):1810-6.

20. Dehaven CD, Evans AM, Dai H, Lawton KA. Organization of GC/MS and LC/MS metabolomics data into chemical libraries. Journal of cheminformatics. 2010;2(1):9.

21. Evans AM, DeHaven CD, Barrett T, Mitchell M, Milgram E. Integrated, nontargeted ultrahigh performance liquid chromatography/electrospray ionization tandem mass spectrometry platform for the identification and relative quantification of the small-molecule complement of biological systems. Analytical chemistry. 2009;81(16):6656-67.

22. Evans AM BB, Liu Q, Mitchell MW, Robinson RJ, Dai H, Stewart SJ, DeHaven CD, Miller LAD. High Resolution Mass Spectrometry Improves Data Quantity and Quality as Compared to Unit Mass Resolution Mass Spectrometry in High-Throughput Profiling Metabolomics. . Metabolomics. 2014;4(1):1-7.

23. Kim DK, Heineman FW, Balaban RS. Effects of beta-hydroxybutyrate on oxidative metabolism and phosphorylation potential in canine heart in vivo. The American journal of physiology. 1991;260(6 Pt 2):H1767-73.

24. Hand DJ. Assessing the performance of classification methods. International Statistical Review. 2012;80(3):400--14.

25. Hastie T, R. Tibshirani and J. H. Friedman The elements of statistical learning : data mining, inference, and prediction. . New York, NY: Springer; 2009.

26. Diaz-Uriarte R, Alvarez de Andres S. Gene selection and classification of microarray data using random forest. BMC bioinformatics. 2006;7:3.

27. Breiman L. Random Forests. Machine Learning 2001;45:5--32.

28. Draper J, Enot DP, Parker D, Beckmann M, Snowdon S, Lin W, et al. Metabolite signal identification in accurate mass metabolomics data with MZedDB, an interactive m/z annotation tool utilising predicted ionisation behaviour 'rules'. BMC bioinformatics. 2009;10:227.

29. Wickham H, SpringerLink (Online service). ggplot2 Elegant Graphics for Data Analysis. New York, NY: Springer-Verlag New York; 2009. Available from: <http://alias.libraries.psu.edu/eresources/proxy/login?url=http://dx.doi.org/10.1007/978-0-387-98141-3>.

30. Darbney A. Storey JD WG. QVALUE: Q-VALUE ESTIMATION FOR FALSE DISCOVERY RATE CONTROL. R PACKAGE VERSION 1360. 2015.

31. Hothorn T, Bretz F, Westfall P. Simultaneous inference in general parametric models. Biometrical journal Biometrische Zeitschrift. 2008;50(3):346-63.

32. Liaw AaW, M. Classification and Regression by randomForest. R News. 2002;2/3:18-22.

33. R WHaF. dplyr: A Grammar of Data Manipulation. R package version 0.5.0. https://CRANR-projectorg/package=dplyr. 2016;Accessed 6/2016.
