# Supplementary figures and images for: Identification of a plasma metabolomic signature of thrombotic myocardial infarction that is distinct from non-thrombotic myocardial infarction and stable coronary artery disease
Source: PLoS One. 2017 Apr 17;12(4):e0175591. doi: 10.1371/journal.pone.0175591 (PMC5393610; doi:10.1371/journal.pone.0175591)

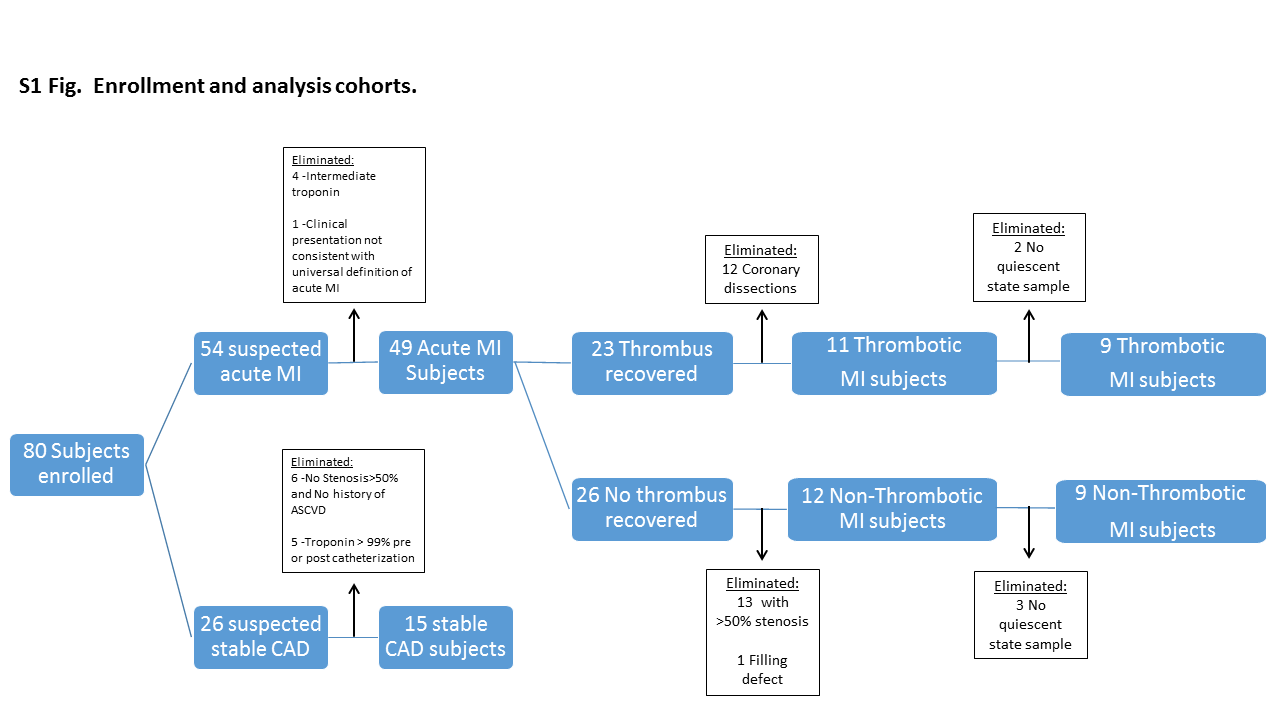

Supplement: S1 Fig — (TIF) [file pone.0175591.s002.tif]

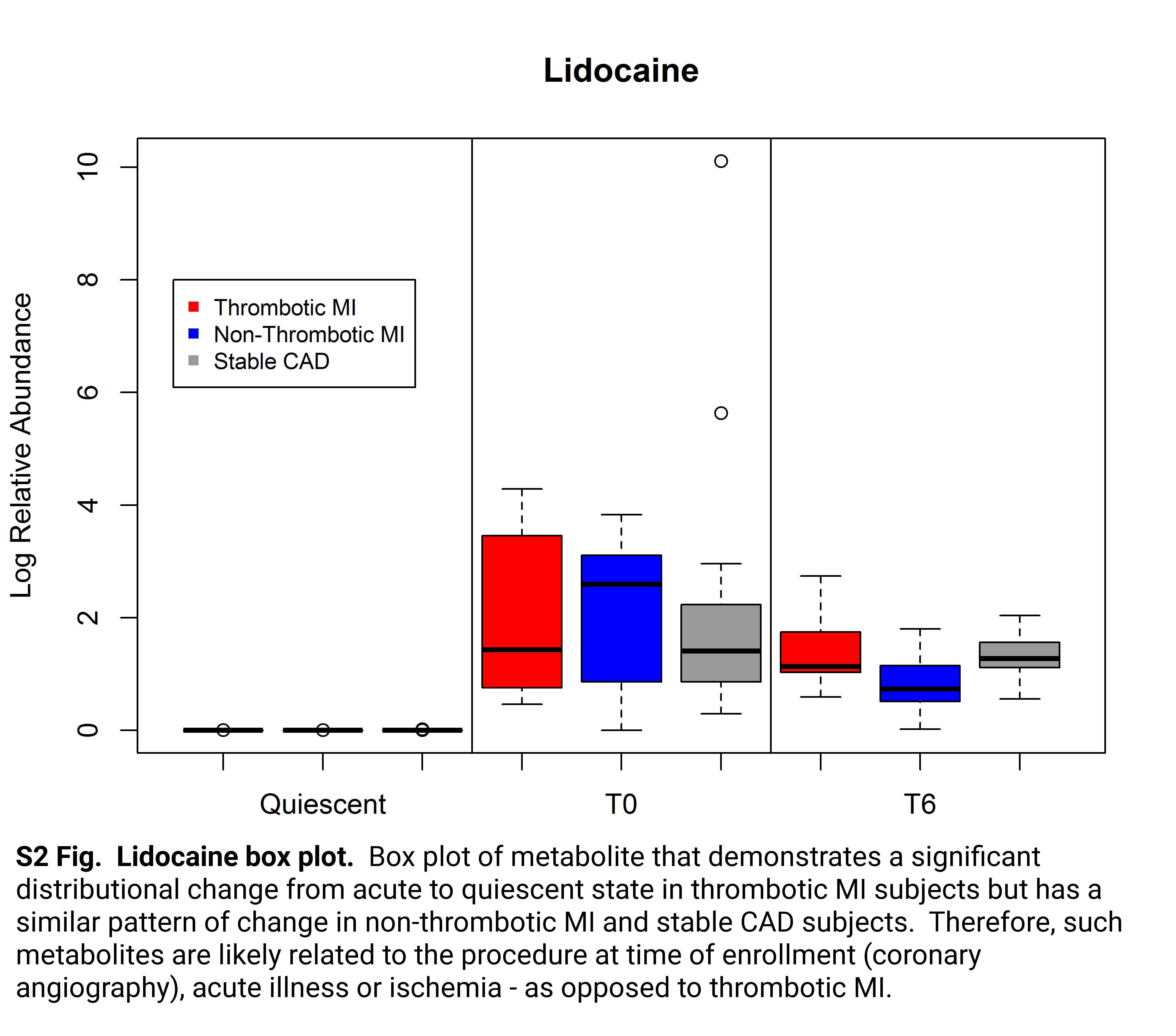

Supplement: S2 Fig — Box plot of metabolite that demonstrates a significant distributional change from acute to quiescent state in thrombotic MI subjects but has a similar pattern of change in non-thrombotic MI and stable CAD subjects. Therefore, such metabolites are likely related to the procedure at time of enrollment (coronary angiography), acute illness or ischemia—as opposed to thrombotic MI. (TIFF) [file pone.0175591.s003.tiff]
